# Role of Exosomal miRNAs in Heart Failure

**DOI:** 10.3389/fcvm.2020.592412

**Published:** 2020-12-17

**Authors:** Ruicong Xue, Weiping Tan, Yuzhong Wu, Bin Dong, Zengshuo Xie, Peisen Huang, Jiangui He, Yugang Dong, Chen Liu

**Affiliations:** ^1^Department of Cardiology, First Affiliated Hospital of Sun Yat-sen University, Guangzhou, China; ^2^NHC Key Laboratory of Assisted Circulation, Sun Yat-sen University, Guangzhou, China; ^3^National-Guangdong Joint Engineering Laboratory for Diagnosis and Treatment of Vascular Diseases, First Affiliated Hospital of Sun Yat-sen University, Guangzhou, China; ^4^Department of Respiratory, the First Affiliated Hospital of Sun Yat-sen University, Guangzhou, China

**Keywords:** heart failure, exosomal miRNAs, cardiomyocyte hypertrophy, cardiac fibrosis, myocardial angiogenesis

## Abstract

Heart failure is the terminal outcome of the majority of cardiovascular diseases, which lacks specific diagnostic biomarkers and therapeutic targets. It contributes to most of cardiovascular hospitalizations and death despite of the current therapy. Therefore, it is important to explore potential molecules improving the diagnosis and treatment of heart failure. MicroRNAs (miRNAs) are small non-coding RNAs that have been reported to be involved in regulating processes of heart failure. After the discovery of miRNAs in exosomes, the subcellular distribution analysis of miRNAs is raising researchers' attention. Growing evidence demonstrates that exosomal miRNAs may be promising diagnostic and therapeutic molecules for heart failure. This review summarizes the role of exosomal miRNAs in heart failure in the prospect of molecular and clinical researches.

## Introduction

Heart failure is the terminal stage of various CVDs, with sustaining high morbidity and mortality contributing most to cardiac death (1). Despite of the current therapy strategy, the mortality and the re-hospitalization of heart failure are still high and no specific drugs are found to reverse heart failure once initiated. Therefore, exploring the pathogenesis of heart failure and labeling molecules closely related to the occurrence and development of heart failure are quite important, since these actions can post significant impact on early detection and treatment of the disease which can be prevented from adverse events.

MicroRNAs (miRNAs) are small non-coding RNAs that have been discovered for over two decades. MiRNAs mainly function as negative regulators of post-transcription of gene expressions through hybridization of 3′ untranslated regions (3′UTRs) or open reading frames (ORFs) of the targeted mRNAs. Although they don't encode any protein, they are widely involved in the modulation of various genes at post-transcription level and thereby impacting various biological processes such as autophagy, oxidative stress and so on (2–5). Exosomes are bilayer membrane extracellular vesicles (EVs) carrying lipids, proteins and nucleic acids. They can be secreted by almost all eukaryotic cells through the fusion of multivesicular bodies and plasma membranes. They are released to extracellular matrix and responsible for carrying biological regulatory information [proteins, lipids, DNAs, and RNAs, especially miRNAs (6)] to the target cells in both surrounding and distant sites and organs, serving as paracrine signaling mediating intercellular communications. Exosomes transport intercellular information via four mechanisms: (1) Exosomal membrane proteins bind to the ligand-receptors in membranes of the target cells directly, activating certain signaling pathways; (2) Exosomal membrane proteins are splintered into pieces, releasing soluble ligands that bind to receptors of recipient cells; (3) Exosomes membranes directly fuse with the membranes of the target cells; (4) Internalization of exosomes by endocytic processes such as phagocytosis and macropinocytosis (7).

MiRNAs distribute widely in cellular components, and recent studies revealed that miRNAs also exist in exosomes (exosomal miRNAs, exo-miRNAs) (8). Furthermore, recent evidences revealed that miRNAs can be isolated easily and steadily from many kinds of tissues and body fluids including circulation, urine and saliva. In circulation, the detective miRNAs are mainly in the forms of exosomal miRNAs. The miRNAs are steady due to the protection of exosomes, whereas miRNAs in free forms will be degraded by nuclease (9). The discovery of exosomal miRNAs especially in circulation broadens functions of miRNAs to intercellular paracrine signaling mediators and disease biomarkers. In heart failure, mounting evidences have suggested that miRNAs participate in its pathogenesis and progression. However, attention focused on the component analysis of miRNAs and the related mechanisms in heart failure are still lacking. This review summarizes the recent evidences and progress of exosomal miRNAs in heart failure.

## Exosomal miRNAs: Origin, Function and Mechanism in CVDs

Exosomal miRNAs are important biological products in exosomes, which are widely involved in information transmission between cells. It is worth noting that miRNAs do not enter exosomes randomly, but specific miRNAs are selectively entered into exosomes. Mature miRNA enters exosomes through four ways: (1) the miRISC related pathway; (2) sphyngomyelinase 2 dependent pathway; (3) heterogeneous nuclear ribonucleoproteins dependent pathway; (4) 3′miRNA sequence-dependent pathway. These are important ways for exosomal miRNAs to achieve directional information exchange between cells. Exosomal miRNAs participate in a variety of basic cell functions through paracrine or endocrine mechanisms, including cell proliferation, apoptosis, cytokine production, immune regulation and metastasis (10, 11). Under pathophysiological conditions, the content of specific exosomal miRNAs will also change (12). This makes evaluating levels of exosomal miRNAs a way for us to diagnose (13–17) and treat diseases (18).

In the cardiovascular field, exosomal miRNAs normally play the role as cardiovascular cells communicators. Almost all kinds of cardiovascular cells are proved to release and receive exosomal miRNAs, indicating their potential regulatory roles in CVDs. Cardiomyocytes and fibroblasts, the most prevalent cell types in the heart, are the main source of exosomal miRNAs. Other than that, ECs and macrophages are also proved to release exosomal miRNAs in different CVDs (19). Depending on the source of exosomal miRNAs, target cells and the disease settings, these exosomal miRNAs act as different regulators on various molecular pathways in different CVDs [such as myocardial infarction, cardiomyopathy, arrhythmia, atherosclerosis and so on (20), Table 1], and the mechanisms involved are various. However, none of the studies successfully illustrate how exosomal miRNAs fulfill their specificity and further illustrated the way specific miRNA incorporate into exosomes. They use exosomes as non-selective transporters rather tissue/cell specific transmitters. Based on the above, we believe that there is still a long way for the research of exosomal miRNAs in CVDs.

**Table 1:** Classification and mechanisms of exosomal miRNAs in CVDs according to the source of cell type.

**Source of exosomes**	**Type of CVD**	**Exo-miRNAs**	**Target cell**	**Target molecular pathway**	**Mechanisms**	**References**
Cardiomyocytes	Chronic heart failure	Exo-miR-217	Cardiac fibroblast	phosphatase and tension homolog deleted on chromosome ten (PTEN)	Induce cardiac fibrosis and cardiac dysfunction	(21)
	Diabetic hearts	Exo-miR-320	ECs	Down-regulated its target genes (IGF-1, Hsp20, and Ets2)	Anti-angiogenic	(22)
ECs	Peripartum cardiomyopathy	Exo-miR-146a	Cardiomyocytes	Downregulation of Erbb4, Nras, Notch1, and Irak1	Decrease metabolic activity	(23)
Cardiac fibroblasts	Cardiac hypertrophy	Exo-miR-21-3p	Cardiomyocytes	Silence sorbin and SH3 domain-containing protein 2 (SORBS2) and PDZ and LIM domain 5 (PDLIM5)	Induce cardiac hypertrophy	(19)
	Atrial fibrillation	Exo-miR-21-3p	Cardiomyocytes	Induce Cav1.2 expression	Increase vulnerability under atrial fibrillation	(24)
Macrophages	Uremic cardiomyopathy	Exo-miR-155	Cardiomyocytes	Repress factors of the O class (FoxO3a)	Induce cardiac hypertrophy and fibrosis	(25)
Fat cells	Cardiac hypertrophy	Exo-miR-200	Cardiomyocytes	Decreased TSC1 and active mTOR	Induce cardiac hypertrophy	(26)
AMSCs	Atrial fibrillation	Exo-miR-320d	Cardiomyocytes	Decreased STAT3-dependent	Induce cardiac hypertrophy	(27)
Unknown	Atherosclerosis	MiR 30e and miR 92a	Unknown	Negative correlate with the plasma ABCA1 level	Might decrease coronary atherosclerosis	(28)

## Exosomal miRNAs in Heart Failure

Heart failure is the terminal stage of almost all cardiovascular disorders mentioned above. It is characterized by a series of pathological processes including cardiomyocyte hypertrophy, cardiac fibrosis, impaired myocardial angiogenesis. Owing to the unique traits of exosomal miRNAs, investigating how they affect the pathogenesis of heart failure is meaningful.

### Exosomal miRNAs in Cardiomyocyte Hypertrophy as Regulators

A series of miRNAs have been demonstrated to modulate the pathogenesis of cardiac hypertrophy which is the key mechanism of heart failure. MiR-199a targets the glycogen synthase kinase 3β (GSK3β)/mammalian target of rapamycin (mTOR) complex signaling pathway to impair cardiomyocyte autophagy and thereby enhancing cardiac hypertrophy (miR-199a impairs autophagy and induces cardiac hypertrophy through mTOR activation). In the model of heart failure, cardiac-specific overexpression of miR-221 significantly deteriorates cardiac function and promotes heart failure and mTOR axis-related autophagy (29). However, few of the studies above have analyzed the component and the origin of the miRNAs involved in the regulation of cardiac hypertrophy. Furthermore, how miRNAs in non-cardiomyocytes exert their anti-hypertrophic effects on cardiomyocytes remains unexplained.

**Figure 1:**
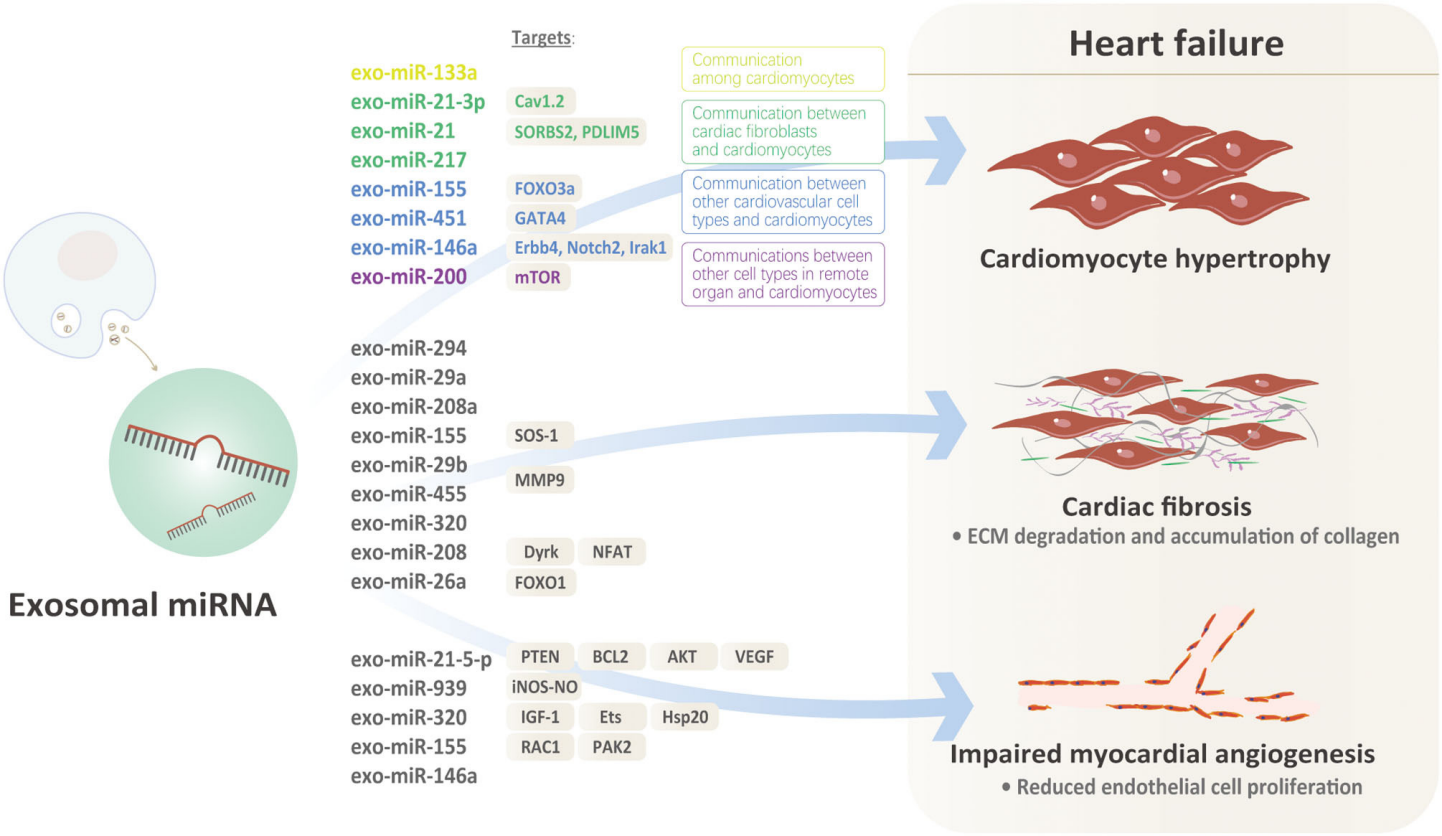
Role of Exosomal miRNA.

Recently, more and more evidences indicate that exosomal miRNAs might illustrate the issues above well Figure 1. Under pro-hypertrophic stresses, exosomal miRNAs from various kinds of cardiovascular cells target at cardiomyocytes, initiating or inhibiting cardiac hypertrophy. Based on the origin of exosomal miRNAs, there are basically three kinds of mechanisms involved: (1) Communication among cardiomyocytes: In MI model, exo-miRNA-133a level was significantly down-regulated in cardiomyocytes in the infarct and peri-infarct areas. Exo-miR-133a released from ischemic cardiomyocytes may be captured by cardiomyocytes adjacent to the nonintact areas, exerting inhibitory effects on hypertrophy by reducing necrosis and apoptosis of cardiomyocytes (30). However, there are no other positive findings that exosomal miRNAs released from cardiomyocytes targeted at cardiomyocytes. We speculate that ligand-receptors for specific exosomal miRNAs from cardiomyocytes are lacking in other cardiomyocytes under the same pathological condition. (2) Communication between cardiac fibroblasts and cardiomyocytes: Cardiac fibroblasts account for ~60–70% of cardiac cells, which makes them sensitive transductors mediating the pro-hypertrophic signals via exosomal-miRNAs. Evidence that miRNA-enriched exosomes are involved in dysregulation of Nrf2-ARE by mediating intercellular communication between fibroblasts and cardiomyocytes supports the communication between cardiac fibroblasts and cardiomyocytes via exosomal miRNAs (31). Clues that murine cardiomyocyte hypertrophy could be induced by co-culturing with cardiac fibroblasts or conditioned fibroblast media further indicates communication between cardiac fibroblasts and cardiomyocyte in cardiac hypertrophy (32, 33). Cardiac fibroblasts secrete exosomes enriched with miR-21-3p, inducing cardiac hypertrophy by targeting recipient cardiomyocytes (19). MiR-21, a proven regulator of fibroblasts biology, is increased in failing hearts, implicating its potential role in heart failure. However, overexpressing or inhibiting miR-21 in cardiomyocytes doesn't influence the hypertrophic phenotype, raising the question that the origin or distribution of miR-21 might be involved. Further evidences suggest that miR-21 expressions are extremely high in exosomes and miR-21 expressions are higher in donor fibroblasts, indicating that miR-21 is packed into exosomes. Furthermore, exo-miR-21 derived from fibroblast is proved to be absorbed into cardiomyocytes and subsequently decreases SORBS2 and PDLIM5 in cardiomyocytes, ultimately inducing cardiac hypertrophy. Thus, exo-miR-21, rather than miR-21, is a key mediator transferring pro-hypertrophy information from cardiac fibroblasts to cardiomyocytes (19). MiR-217 was also proved to be a potent marker of chronic heart failure, evidenced by increased expressions in heart failure and its overexpression pressure overload-induced cardiac hypertrophy. Further component analysis proved that cardiomyocyte-derived exosomes containing miR-217 enhanced proliferation of fibroblasts *in vitro*, suggesting that exosomal miR-217 was not only a potent marker but a promising therapeutic target for chronic heart failure (21). (3) Communications between other cardiovascular cell types and cardiomyocytes: Exosomes enriched with miR-155 which were derived from macrophages promoted cardiac hypertrophy and fibrosis by activating the pro-hypertrophic pathway FOXO3a in uremic mice (25). In acute MI, exosomal miRNAs also acted in cell-cell communication between cardiomyocytes and local or remote stem cells. For example, exosomes enriched with miR-451 that derived from cardiac progenitor cells protected cardiomyocytes from oxidative stress by targeting GATA4, thereby maintaining cardiac function. Exo-miR-146a extracted from ECs were absorbed by cardiomyocytes and subsequently targeted on Erbb4, Notch2, Irak1, mediating the progression of heart failure through metabolic pathways (23). (4) Communications between other cell types in remote organs and cardiomyocytes: A recent research proved that PPAR-γ activation in adipocytes from adipose tissues increased the secretion of miRNA-200-contained exosomes which promoted cardiomyocyte hypertrophy through the mTOR pathway (26).

Exosomal miRNAs are involved in the regulation of cardiac hypertrophy due to various etiology. However, based on the current evidences, the defined role of exosomal miRNAs in cardiac hypertrophy differs across disease settings and the sources, not to mention the miscellaneous mechanisms involved. We still lack further research illustrating how exosomes ensure the specificity for certain miRNAs targeting at specific cell types.

### Exosomal miRNAs in Cardiac Fibrosis as Regulators

Cardiac fibrosis, characterized by ECM degradation and accumulation of collagen due to proliferation of cardiac fibroblasts, is another important mechanism of heart failure. MiRNAs have been reported to regulate cardiac fibrosis. For example, miR-19a-3p and miR-19b-3p targeted TGF-β to inhibit epithelial-mesenchymal transition, ECM production and the invasion of human cardiac fibroblasts, thereby exhibiting the anti-fibroblast effect (34). More lights are shed on the regulation of cardiac fibrosis by exosomal miRNAs. Exosomal miR-294 from embryonic stem cells were proved to inhibit fibrosis after MI, thereby preventing MI-induced heart failure. Elevated exo-miR-29a in marginal ischemic area transferred the intercellular information among cardiomyocytes, mediating the antifibrotic effect and preventing ventricular dysfunction and heart failure (35). MiR-208a was upregulated in cardiomyocytes and cardiomyocyte-derived exosomes in cardiac fibrosis (36).

Mechanically, exosomal miRNAs from diverse sources participate in the regulation of different parts of cardiac fibrosis Figure 1. Macrophage-derived exo-miR-155 inhibited the proliferation of cardiac fibroblasts after MI by inhibiting the SOS-1, a critical modulator of RAS activation in mice (37). Cardiomyocyte-derived exosomes containing miR-208a contributed to increased fibroblast proliferation and differentiation into myofibroblasts and thereby accelerating the progression of cardiac fibrosis (36). In type 2 diabetes mice, exercise significantly increased exosomes derived from cardiomyocytes which contain abundant miR-29b and miR-455, thereby decreasing MMP9 and ECM degradation and resulting in the amelioration of cardiac fibrosis (38). In diabetic cardiomyopathy, exosomes derived from diabetic cardiomyocytes negatively affected proliferation and migration of ECs by transferring miRNA-320 and subsequently inhibited heart failure. This process was reversed by adding GW-4869, an exosome inhibitor (22). Exosomal miR-208 derived from cardiomyocytes inhibited the expression of Dyrk and phosphorylated NFAT to promote its nuclear export. Nuclear NFAT triggered fibrosis by inducing the expression of CTGF, Col1a1, Col3a1, and a-SMA in cardiac fibroblasts (36). The evidences above have proved the fact that miRNAs in exosomes are important regulators of cardiac fibrosis and heart failure due to different etiologies. Injection of exo-miR-26a into tibialis anterior (TA) muscle significantly increased the expression of miR-26a in the heart and subsequently ameliorated cardiac fibrosis lesions. Furthermore, exo-miR-26a significantly blunted FOXO1 activation and inhibited cardiac fibrosis in the heart of mice with chronic kidney disease (39). This study demonstrated the strength of miRNAs encapsulated by exosomes for its tissue specificity, however, how exosomes guarantee this specificity needs illustrating. Furthermore, whether this specificity was due to the encapsulation of exosomes or miRNAs themselves are yet unknown.

### Exosomal miRNAs in Impaired Myocardial Angiogenesis as Regulators

Besides of the pathologic changes in heart tissue, aberrant angiogenesis contributes to the acceleration from compensated cardiac hypertrophy to heart failure due to decreasing capillary angiogenesis and cardiomyocyte energy supply (40). ECs proliferation is the key factor in this process (41). Exosomal miRNAs have been proved to regulate angiogenesis in other diseases Figure 1, but how they affect angiogenesis in heart failure is not fully and directly illustrated.

Exo-miR-21-5-p from healthy hearts inhibited PTEN and BCL2 in cardiomyocytes and ECs and activated the AKT and VEGF pathways to promote cardiomyocyte proliferation and promote angiogenesis (42). Exosomes containing miR-939 from hearts in patients with myocardial ischemia significantly promoted angiogenesis through iNOS-NO pathway (43). However, the source of exosomal miRNAs in the researches above might need further study. In the subsequent studies, they perfectly demonstrated the sources, target cells and the mechanisms of exosomal miRNAs in the regulation of angiogenesis. Wang et al. discovered that exosomes isolated from diabetic cardiomyocytes significantly reduced angiogenesis in the diabetic myocardium via the exosomal transfer of miR-320 into ECs and subsequent decrease of IGF-1, Ets and Hsp20 (22). In MI, M1-like macrophages released a large number of pro-inflammatory exosomes, transferring miR-155 to ECs to inhibit angiogenesis by down-regulating Rac family small GTPase 1 and p21 activates kinase 2 (44). Interestingly, we discovered the same miRNA might exert completely opposite regulatory effects in angiogenesis in the process of heart failure. MiR-146a in the exosomes derived from ECs significantly decreased ECs proliferation and inhibited microvascular regeneration and impaired systolic/diastolic function in cardiomyopathy (23). However, exosomal miR-146a derived from cardiosphere cells promoted myocardial angiogenesis and significantly improved heart failure after acute MI (45). Some scholars believe that these contradictions may be related to different periods of heart failure. In the early stage, cardiac exosomes transmitted cardioprotective miRNAs, and in the terminal stages, exosomal miRNAs may play a damaging role (46). In addition, we also assume that the seemingly opposite results might be attributed to the origin of miRNA and different etiologies of heart failure. Further mechanistic research demonstrating this contradiction is required.

## Application of Exosomal miRNAs in Heart Failure: Biomarkers and Therapeutic Targets

The exploration of new biomarkers of heart failure is critical for the diagnosis and treatment of heart failure. BNP and NT-proBNP are widely used in the clinical diagnosis of heart failure. Some newly found biomarkers such as ST2 and NGAL also become to be used in clinical. However, in the case of combination with some other pathological conditions, such as pulmonary embolism and renal dysfunction, the specificity of the biomarkers mentioned above could be compromised.

A variety of miRNAs in circulation are involved in pathogenesis or protection of heart failure in a dynamic and stage-specific way and therefore they are potential biomarkers of heart failure. Circulating miR-23a, miR-23b, miR-24, miR-195, and miR-214 were found increased and circulating miR-423-5p, miR-320, and miR-22 were found decreased in chronic heart failure (47, 48). As for acute heart failure (AHF), miR-423-5p was proved to differentially express between heart failure patients and controls, which might serve as a biomarker distinguishing AHF and other causes of dyspnea (49). MiR-210 in plasma was also proved to be elevated in heart failure patients with NYHA III-IV compared with those with NYHA II and controls (50). Other differential expression circulating miRNAs in AHF includes down-regulated miR-103, miR-142-3p, miR-30b, miR-342-3p and up-regulated miR-499 (51, 52). Furthermore, miRNAs were also proved to have potential ability to evaluate prognosis. Decreased levels of miR-18a-5p and miR-652-3p during hospitalization in heart failure patients were associated with a high predicted mortality within 180 days (53). Up-regulation of miR-192 was proved to be a prognostic marker predicting survival in ischemic heart failure with p53-mediated apoptosis involved (54).

However, none of the studies above analyzed the circulating components, especially exosome fraction and non-exosome supernatant. Wang et al. discovered that miR-425 and miR-744 expressions were altered in plasma exosome samples in heart failure patients. A further functional study proved that they negatively regulated cardiac fibrosis by suppressing TGFβ1 expression, revealing their potential role in regulating and predicting heart failure (55). However, direct evidence of the regulatory role of miRNAs via exosomes was lacking in this study. Circulating miRNAs exist in the form of free miRNAs and exosome-encapsulated miRNAs. Free miRNAs in circulation are the byproduct of dead cells and their levels might be altered depending on the rates of tissue repair and age. On the contrary, exosomal miRNAs, making up 10–15% of circulating miRNAs, are more sensitive in providing stable and accurate patterns for disease diagnosis. However, the difficulty to isolate and detect circulating exosomal miRNAs limited their further application as heart failure biomarkers. As the technology develops, Turchinovich et al. firstly separated non-exosome supernatant fraction from exosome and Beg et al. constructed protocols to measure both free miRNAs and exosomal miRNAs in circulation, making it possible to proceed the biomarkers exploration focusing on exosomal miRNAs (56, 57). In the study of Beg's, they found an increase of miR-146a and miR-486 (both had the poverty of anti-inflammation) in exosomes rather than those in plasma in the patients with heart failure compared to controls, indicating that exosomal miRNAs worth being further studied as heart failure biomarkers. Inspiringly, two studies supported the diagnostic value of exosomal miRNAs. Serum exo-miR-92b-5p was proved to be increased in HFrEF patients compared with controls, with a sensitivity of 71.4% and specificity of 83.3%. Moreover, exo-miR-92b-5p was inversely correlated with the LVFS and LVEF, while positively correlated with LAD, LVDD and LVSD. Serum exo-miR-92b-5p is a potential diagnostic biomarker in AHF due to DCM, the elevation of which indicated the progression of AHF (58). In another study focused on ischemic HF post AMI discovered that both serum levels and exosomal levels of miR-192, miR-194, and miR-34a were up-regulated in HF patients, confirming their diagnostic role in ischemic HF (59). However, this study did not compare the disparity between serum miRNAs and exosomal miRNAs regarding to their diagnostic value. In myxomatous mitral valve disease model of dogs, decreased exosomal miR-599 levels and increased exosomal miR-181c and miR-495 were found in chronic heart failure compared to controls, whereas no miRNA in total plasma remained statistically significant alteration if the false discovery rate was set <15%, indicating that exosomal miRNAs might be a more specific marker than total plasma miRNAs (60). The concern of the comparation of the diagnostic value between exosomal miRNAs and free miRNAs are raised consequently. Some studies supported that exosomal miRNAs exhibit more diagnostic and prognostic value than those in free form. For example, in stable coronary artery heart disease, miR-126 and miR-199a in exosomes were proved to be related with cardiovascular events rather than free miR-126 and miR-199a in plasma (61). However, a study opposed this opinion with the evidence that no differential expression of exosomal miRNA and free-miRNA in circulation were detected between chronic heart failure patients and controls (48). In a 30 patients' clinical study, the disparity of miRNA levels (miR-423-5p/320a/22/92b/17/532-3p/92a/30a/21/101) between patients with heart failure and controls were similar in exosomal fraction and unfractionated serum, suggesting that these miRNAs in exosome might not be ideal markers for heart failure. However, we doubt that the sample capacity in this study was small and the coverage of miRNA detected was limited (48). Further studies are desperately needed to demonstrate the diagnostic value of exosomal miRNAs in heart failure by expanding the number of patients enrolled and the miRNAs. Furthermore, the source of most of the exosomal miRNAs detected in circulation has not been further illustrated, limiting the mechanism exploration of the specific exosomal miRNAs.

## Stem Cells-Derived Exosomal miRNAs in Heart Failure: Opportunities and Challenges

The stem cell-based therapies are becoming potential therapeutic strategies in many diseases and their influences radiate to heart failure, whereas the low efficiency of engraftment and potential tumorigenic risk of stem cell transplantation limits their prevalence. However, the emerging value of stem cell-derived exosomes which might improve the efficiency of stem cell transplantation motivates the further exploration of stem cell-based therapies in heart failure. Exosomes derived from different kinds of stem cells, such as ESCs, iPSCs CPCs and MSCs, have been proved to exert no less protective properties but safer in operation than stem cell transplantation (62). As one of the major components in exosomes, several miRNAs in exosomes derived from stem cells have been identified to play important roles in heart failure. Intramyocardial delivery of exosomes enriched with miR-290-295 and miR-294 derived from ESCs significantly ameliorated fibrosis in acute MI. Exosomes from murine cardiac fibroblast-derived iPSCs, which were enriched with miR-21 and miR-210, have been proved to protect against oxidative stress and cardiac remodeling after acute MI in peri-infarct regions. And the protective properties of miR-21 enriched exosomes were similar with miR-21 (63, 64). CPCs treatments help to improve cardiac repair and protect against damage and exosomes in CPCs are involved. Proangiogenic miR-17 and miR-210 were up-regulated in CPC-derived exosomes under oxidative stress and thereby inhibited TGF-β induced fibrosis, providing another profound evidence that miRNAs in exosomes might be a pivotal component in the stem cell therapy of heart failure (65). Researchers found that bone marrow-derived MSC (BMSC) from mice after ischemic preconditioning could secrete miR-22 highly enriched exosomes, and in the MI mouse model, miR-22 targeted methyl CpG binding protein 2 (Mecp2) to reduce infarct size and cardiac fibrosis (66). After treating human engineered cardiac tissue (hECT) with exosomes derived from human mesenchymal stem cells (hMSCs), the level of miR-21-5p is significantly increased in hECT, while knockout of miR-21-5p in hMSCs reduced expression of pro-contraction and related calcium-treated genes in the hECT (67). These findings indicate that stem cell-derived exosomes contain a variety of miRNAs which play an important role in heart failure (Table 2). The use of these exosomes may contribute to the development of novel treatment strategies for heart failure in the future.

**Table 2:** The role of miRNAs in stem cell-derived exosomes in heart failure.

**MiRNA**	**Sources**	**Animal model**	**Function**	**References**
miR-290-295	ESC	AMI	Ameliorated fibrosis in acute MI	(68)
miR-294	ESC	AMI	Ameliorated fibrosis in acute MI	(68)
miR-21	iPSC	MI/R	Protect against oxidative stress and cardiac remodeling after acute MI/R	(64)
miR-210	iPSC	MI/R	Protect against oxidative stress and cardiac remodeling after acute MI/R	(64)
miR-17	CPC	MI/R	Inhibited fibrosis	(65)
miR-210	CPC	MI/R	Inhibited fibrosis	(65)
miR-22	BMSC	MI/R	Reduce infarct size and cardiac fibrosis	(66)
miR-21-5p	hMSC	hECT	Promotes contraction and calcium processing	(67)

## Prospect

The types and levels of miRNAs in exosomes in the circulatory system are more closely related to heart failure compared to free miRNAs. Other than free miRNAs, their level changes may be related to the severity of heart failure. So, they can provide a more sensitive and specific diagnostic method for heart failure. However, there is evidence that no differential expression of exosomal miRNAs and free miRNAs in the circulation was detected between patients with chronic heart failure and the controls. Therefore, whether circulating exosomes miRNAs can be used as biomarkers for the diagnosis and prognosis of heart failure is still questionable. We need studies with larger number of patients and the kinds of miRNAs to determine: whether there are significant changes in the levels of circulating exosomal miRNA after heart failure, whether there is a correlation between these changes and the pathophysiological changes in heart failure, whether the sensitivity and specificity of these new diagnostic methods are better than those currently used in clinical practice.

Heart failure may affect circulating exosomal miRNAs, and exosomes derived from stem cells can also play an important role in heart failure. They may serve as a new strategy for treating or preventing heart failure in the future. However, this new strategy is in its infancy. There are still several hurdles before achieving clinical application (62): First of all, the purification and separation technology of exosomes is not perfect, it takes a long time and the efficiency is low (69). Secondly, exosomes contain a large number of biologically active factors, so they may cause adverse or side effects on cardiac tissue. Thirdly, due to the heterogeneous components in exosomes, it may show a potential risk for tumor formation and a risk of adverse immune response based on donor cell characteristics. Finally, due to the complex structure of exosomes, the therapeutic effect that exosomes produced during the disease is unknown. How exosomal miRNAs fulfill its specificity is yet fully understood. Therefore, the future clinical translation of exosomes for heart failure treatment still faces major challenges. We need to clearly analyze the various components in stem-derived exosomes, clarifying their physiological effects on cardiac tissue, as well as their therapeutic and side effects on human body.

## Author Contributions

RX, ZX, PH, and WT wrote the manuscript. YW drew the figure. JH, CL, and YD edited the manuscript. All authors contributed to the article and approved the submitted version.

## Conflict of Interest

The authors declare that the research was conducted in the absence of any commercial or financial relationships that could be construed as a potential conflict of interest.

## Abbreviations

miRNAs: MicroRNAs
3′UTRs: 3′ untranslated regions
ORFs: Open reading frames
EVs: Extracellular vesicles
ECs: Endothelial cells
CVDs: Cardiovascular diseases
MI: Myocardial infarction
ECM: Extracellular matrix
MMP: Matrix Metalloproteinases
GSK3β: Glycogen synthase kinase 3β
mTOR: Mammalian target of rapamycin
Nrf2: Nuclear factor erythroid2-related factor 2
ARE: Antioxidant response elements
SOS-1: Son of Sevenless-1
Dyrk: Dual-specificity tyrosine-regulated kinases
CTGF: Connective Tissue Growth Factor
TA: Tibialis anterior
AHF: Acute heart failure
HFrEF: Heart failure with reduced ejection fraction
LVFS: Left ventricular fraction shortening
LVEF: Left ventricular ejection fraction
LAD: Left atrial diameter
LVDD: Left ventricular diastolic diameters
LVSD: Left ventricular systolic diameters
DCM: Dilated cardiomyopathy
ESCs: Embryonic stem cells
iPSCs: Induced pluripotent stem cells
CPCs: Cardiac progenitor cells
MSCs: Mesenchymal stem cells
BMSC: Bone marrow-derived MSC
hECT: Human engineered cardiac tissue
hMSCs: Human mesenchymal stem cells.

## References

[1] Berezin A. Epigenetics in heart failure phenotypes. BBA Clin. (2016) 6:31–7. 10.1016/j.bbacli.2016.05.005 27335803 PMC4909708

[2] Huang Z Wu S Kong F Cai X Ye B Shan P. MicroRNA-21 protects against cardiac hypoxia/reoxygenation injury by inhibiting excessive autophagy in H9c2 cells via the Akt/mTOR pathway. J Cell Mol Med. (2017) 21:467–74. 10.1111/jcmm.12990 27680680 PMC5323864

[3] Xu C Hu Y Hou L Ju J Li X Du N. β-Blocker carvedilol protects cardiomyocytes against oxidative stress-induced apoptosis by up-regulating miR-133 expression. J Mol Cell Cardiol. (2014) 75:111–21. 10.1016/j.yjmcc.2014.07.009 25066695

[4] Li J Donath S Li Y Qin D Prabhakar BS Li P. miR-30 regulates mitochondrial fission through targeting p53 and the dynamin-related protein-1 pathway. PLoS Genet. (2010) 6:e1000795. 10.1371/annotation/4050116d-8daa-4b5a-99e9-34cdd13f6a26 20062521 PMC2793031

[5] Khani P Nasri F Khani Chamani F Saeidi F Sadri Nahand J Tabibkhooei A. Genetic and epigenetic contribution to astrocytic gliomas pathogenesis. J Neurochem. (2019) 148:188–203. 10.1111/jnc.14616 30347482

[6] Meng Y Sun J Wang X Hu T Ma Y Kong C. Exosomes: a promising avenue for the diagnosis of breast cancer. Technol Cancer Res Treat. (2019) 18:1077089069. 10.1177/1533033818821421 30760122 PMC6373987

[7] Turturici G Tinnirello R Sconzo G Geraci F. Extracellular membrane vesicles as a mechanism of cell-to-cell communication: advantages and disadvantages. Am J Physiol. (2014) 306:C621–33. 10.1152/ajpcell.00228.2013 24452373

[8] Aghabozorgi AS Ahangari N Eftekhaari TE Torbati PN Bahiraee A Ebrahimi R. Circulating exosomal miRNAs in cardiovascular disease pathogenesis: new emerging hopes. J Cell Physiol. (2019) 234:21796–809. 10.1002/jcp.28942 31273798

[9] Zampetaki A Willeit P Drozdov I Kiechl S Mayr M. Profiling of circulating microRNAs: from single biomarkers to re-wired networks. Cardiovasc Res. (2012) 93:555–62. 10.1093/cvr/cvr266 22028337 PMC3291086

[10] Sadri Nahand J Moghoofei M Salmaninejad A Bahmanpour Z Karimzadeh M Nasiri M. Pathogenic role of exosomes and microRNAs in HPV-mediated inflammation and cervical cancer: a review. Int J Cancer. (2020) 146:305–20. 10.1002/ijc.32688 31566705 PMC6999596

[11] Huang-Doran I Zhang C Vidal-Puig A. Extracellular vesicles: novel mediators of cell communication in metabolic disease. Trends Endocrinol Metab. (2017) 28:3–18. 10.1016/j.tem.2016.10.003 27810172

[12] Zhang J Li S Li L Li M Guo C Yao J. Exosome and exosomal microRNA: trafficking, sorting, and function. Genom Proteom Bioinform. (2015) 13:17–24. 10.1016/j.gpb.2015.02.001 25724326 PMC4411500

[13] Nahand JS Mahjoubin-Tehran M Moghoofei M Pourhanifeh MH Mirzaei HR Asemi Z. Exosomal miRNAs: novel players in viral infection. Epigenomics. (2020) 12:353–70. 10.2217/epi-2019-0192 32093516 PMC7713899

[14] Ghaemmaghami AB Mahjoubin-Tehran M Movahedpour A Morshedi K Sheida A Taghavi SP. Role of exosomes in malignant glioma: microRNAs and proteins in pathogenesis and diagnosis. Cell Comm Signal. (2020) 18:120. 10.1186/s12964-020-00623-9 32746854 PMC7397575

[15] Aghdam AM Amiri A Salarinia R Masoudifar A Ghasemi F Mirzaei H. MicroRNAs as diagnostic, prognostic, and therapeutic biomarkers in prostate cancer. Crit Rev Eukar Gene. (2019) 29:127–39. 10.1615/CritRevEukaryotGeneExpr.2019025273 31679268

[16] Pourhanifeh MH Mahjoubin-Tehran M Karimzadeh MR Mirzaei HR Razavi ZS Sahebkar A. Autophagy in cancers including brain tumors: role of MicroRNAs. Cell Comm Signal. (2020) 18:88. 10.1186/s12964-020-00587-w 32517694 PMC7285723

[17] Hashemian SM Pourhanifeh MH Fadaei S Velayati AA Mirzaei H Hamblin MR. Non-coding RNAs and exosomes: their role in the pathogenesis of sepsis. Mol Therapy Nucleic Acids. (2020) 21:51–74. 10.1016/j.omtn.2020.05.012 32506014 PMC7272511

[18] Rezaei S Mahjoubin-Tehran M Aghaee-Bakhtiari SH Jalili A Movahedpour A Khan H. Autophagy-related MicroRNAs in chronic lung diseases and lung cancer. Crit Rev Oncol Hematol. (2020) 153:103063. 10.1016/j.critrevonc.2020.103063 32712519

[19] Bang C Batkai S Dangwal S Gupta SK Foinquinos A Holzmann A. Cardiac fibroblast-derived microRNA passenger strand-enriched exosomes mediate cardiomyocyte hypertrophy. J Clin Investig. (2014) 124:2136–46. 10.1172/JCI70577 24743145 PMC4001534

[20] Colpaert RMW Calore M. MicroRNAs in cardiac diseases. Cells Basel. (2019) 8:737. 10.3390/cells8070737 31323768 PMC6678080

[21] Nie X Fan J Li H Yin Z Zhao Y Dai B. miR-217 promotes cardiac hypertrophy and dysfunction by targeting PTEN. Mol Therapy Nucleic Acids. (2018) 12:254–66. 10.1016/j.omtn.2018.05.013 30195764 PMC6005806

[22] Wang X Huang W Liu G Cai W Millard RW Wang Y. Cardiomyocytes mediate anti-angiogenesis in type 2 diabetic rats through the exosomal transfer of miR-320 into endothelial cells. J Mol Cell Cardiol. (2014) 74:139–50. 10.1016/j.yjmcc.2014.05.001 24825548 PMC4120246

[23] Halkein J Tabruyn SP Ricke-Hoch M Haghikia A Nguyen N Scherr M. MicroRNA-146a is a therapeutic target and biomarker for peripartum cardiomyopathy. J Clin Investig. (2013) 123:2143–54. 10.1172/JCI64365 23619365 PMC3638905

[24] Li S Gao Y Liu Y Li J Yang X Hu R. Myofibroblast-derived exosomes contribute to development of a susceptible substrate for atrial fibrillation. Cardiology. (2020) 145:324–32. 10.1159/000505641 32235120

[25] Wang B Wang Z Ji J Gan W Zhang A Shi H. Macrophage-derived exosomal Mir-155 regulating cardiomyocyte pyroptosis and hypertrophy in uremic cardiomyopathy. JACC. (2020) 5:148–66. 10.1016/j.jacbts.2019.10.011 32140622 PMC7046511

[26] Fang X Stroud MJ Ouyang K Fang L Zhang J Dalton ND. Adipocyte-specific loss of PPARγ attenuates cardiac hypertrophy. JCI Insight. (2016) 1:e89908. 10.1172/jci.insight.89908 27734035 PMC5053146

[27] Liu L Zhang H Mao H Li X Hu Y. Exosomal miR-320d derived from adipose tissue-derived MSCs inhibits apoptosis in cardiomyocytes with atrial fibrillation (AF). Artif Cells Nanomed Biotechnol. (2019) 47:3976–84. 10.1080/21691401.2019.1671432 31591913

[28] Wang Z Zhang J Zhang S Yan S Wang Z Wang C. MiR-30e and miR-92a are related to atherosclerosis by targeting ABCA1. Mol Med Rep. (2019) 19:3298–304. 10.3892/mmr.2019.9983 30816508

[29] Su M Wang J Wang C Wang X Dong W Qiu W. MicroRNA-221 inhibits autophagy and promotes heart failure by modulating the p27/CDK2/mTOR axis. Cell Death Differ. (2015) 22:986–99. 10.1038/cdd.2014.187 25394488 PMC4423182

[30] Kuwabara Y Ono K Horie T Nishi H Nagao K Kinoshita M. Increased microRNA-1 and microRNA-133a levels in serum of patients with cardiovascular disease indicate myocardial damage. Circ Cardiovasc Genet. (2011) 4:446–54. 10.1161/CIRCGENETICS.110.958975 21642241

[31] Tian C Gao L Zimmerman MC Zucker IH. Myocardial infarction-induced microRNA-enriched exosomes contribute to cardiac Nrf2 dysregulation in chronic heart failure. Am J Physiol. (2018) 314:H928–39. 10.1152/ajpheart.00602.2017 29373037 PMC6008149

[32] LaFramboise WA Scalise D Stoodley P Graner SR Guthrie RD Magovern JA. Cardiac fibroblasts influence cardiomyocyte phenotype in vitro. Am J Physiol. (2007) 292:C1799–808. 10.1152/ajpcell.00166.2006 17229813

[33] Fredj S Bescond J Louault C Potreau D. Interactions between cardiac cells enhance cardiomyocyte hypertrophy and increase. J Cell Physiol. (2005) 202:891–9. 10.1002/jcp.20197 15389635

[34] Zou M Wang F Gao R Wu J Ou Y Chen X. Autophagy inhibition of hsa-miR-19a-3p/19b-3p by targeting TGF-β R II during TGF-β1-induced fibrogenesis in human cardiac fibroblasts. Sci Rep. (2016) 6:24747. 10.1038/srep24747 27098600 PMC4838850

[35] Yamaguchi T Izumi Y Nakamura Y Yamazaki T Shiota M Sano S. Repeated remote ischemic conditioning attenuates left ventricular remodeling via exosome-mediated intercellular communication on chronic heart failure after myocardial infarction. Int J Cardiol. (2015) 178:239–6. 10.1016/j.ijcard.2014.10.144 25464262

[36] Yang J Yu X Xue F Li Y Liu W Zhang S. Exosomes derived from cardiomyocytes promote cardiac fibrosis via myocyte-fibroblast cross-talk. Am J Transl Res. (2018) 10:4350–66. 30662677 PMC6325490

[37] Wang C Zhang C Liu LAX Chen B Li Y. Macrophage-derived mir-155-containing exosomes suppress fibroblast proliferation and promote fibroblast inflammation during cardiac injury. Mol Therapy. (2017) 25:192–204. 10.1016/j.ymthe.2016.09.001 28129114 PMC5363311

[38] Chaturvedi P Kalani A Medina I Familtseva A Tyagi SC. Cardiosome mediated regulation of MMP9 in diabetic heart: role of mir29b and mir455 in exercise. J Cell Mol Med. (2015) 19:2153–61. 10.1111/jcmm.12589 25824442 PMC4568920

[39] Wang B Zhang A Wang H Klein JD Tan L Wang Z. miR-26a limits muscle wasting and cardiac fibrosis through exosome-mediated microRNA transfer in chronic kidney disease. Theranostics. (2019) 9:1864–77. 10.7150/thno.29579 31037144 PMC6485283

[40] Gogiraju R Bochenek ML Schäfer K. Angiogenic endothelial cell signaling in cardiac hypertrophy and heart failure. Front Cardiovasc Med. (2019) 6:20. 10.3389/fcvm.2019.00020 30895179 PMC6415587

[41] Zhabyeyev P Gandhi M Mori J Basu R Kassiri Z Clanachan A. Pressure-overload-induced heart failure induces a selective reduction in glucose oxidation at physiological afterload. Cardiovasc Res. (2013) 97:676–85. 10.1093/cvr/cvs424 23257023

[42] Qiao L Hu S Liu S Zhang H Ma H Huang K. microRNA-21-5p dysregulation in exosomes derived from heart failure patients impairs regenerative potential. J Clin Investig. (2019) 129:2237–50. 10.1172/JCI123135 31033484 PMC6546482

[43] Li H Liao Y Gao L Zhuang T Huang Z Zhu H. Coronary serum exosomes derived from patients with myocardial ischemia regulate angiogenesis through the miR-939-mediated nitric oxide signaling pathway. Theranostics. (2018) 8:2079–93. 10.7150/thno.21895 29721064 PMC5928872

[44] Liu S Chen J Shi J Zhou W Wang L Fang W. M1-like macrophage-derived exosomes suppress angiogenesis and exacerbate cardiac dysfunction in a myocardial infarction microenvironment. Basic Res Cardiol. (2020) 115:22. 10.1007/s00395-020-0781-7 32112145

[45] Ibrahim AG Cheng K Marbán E. Exosomes as critical agents of cardiac regeneration triggered by cell therapy. Stem Cell Rep. (2014) 2:606–19. 10.1016/j.stemcr.2014.04.006 24936449 PMC4050492

[46] Ribeiro-Rodrigues TM Laundos TL Pereira-Carvalho R Batista-Almeida D Pereira R Coelho-Santos V. Exosomes secreted by cardiomyocytes subjected to ischaemia promote cardiac angiogenesis. Cardiovasc Res. (2017) 113:1338–50. 10.1093/cvr/cvx118 28859292

[47] Pfeifer P Werner N Jansen F. Role and function of microRNAs in extracellular vesicles in cardiovascular biology. Biomed Res Int. (2015) 2015:161393. 10.1155/2015/161393 26558258 PMC4618108

[48] Goren Y Kushnir M Zafrir B Tabak S Lewis BS Amir O. Serum levels of microRNAs in patients with heart failure. Eur J Heart Fail. (2012) 14:147–54. 10.1093/eurjhf/hfr155 22120965

[49] Schneider SIDR Silvello D Martinelli NC Garbin A Biolo A Clausell N. Plasma levels of microRNA-21,−126 and−423-5p alter during clinical improvement and are associated with the prognosis of acute heart failure. Mol Med Rep. (2018) 17:4736–46. 10.3892/mmr.2018.8428 29344661

[50] Endo K Naito Y Ji X Nakanishi M Noguchi T Goto Y. MicroRNA 210 as a biomarker for congestive heart failure. Biol Pharm Bull. (2013) 36:48–54. 10.1248/bpb.b12-00578 23302636

[51] Ellis KL Cameron VA Troughton RW Frampton CM Ellmers LJ Richards AM. Circulating microRNAs as candidate markers to distinguish heart failure in breathless patients. Eur J Heart Fail. (2013) 15:1138–47. 10.1093/eurjhf/hft078 23696613

[52] Corsten MF Dennert R Jochems S Kuznetsova T Devaux Y Hofstra L. Circulating microRNA-208b and microRNA-499 reflect myocardial damage in cardiovascular disease. Circ Cardiovasc Genet. (2010) 3:499–506. 10.1161/CIRCGENETICS.110.957415 20921333

[53] Ovchinnikova ES Schmitter D Vegter EL Ter Maaten JM Valente MAE Liu LCY. Signature of circulating microRNAs in patients with acute heart failure. Eur J Heart Fail. (2016) 18:414–23. 10.1002/ejhf.332 26345695

[54] Stefanie K Sebastian E Jürgen P Till N Michael A Frey UH. Circulating miR-192 is a prognostic marker in patients with ischemic cardiomyopathy. Fut Cardiol. (2018) 14:283–9. 10.2217/fca-2017-0108 29927310

[55] Lu W Jiao L Bin X Yu-Lan L Zhou L. Reduced exosome miR-425 and miR-744 in the plasma represents the progression of fibrosis and heart failure. Kaohsiung J Med Sci. (2018) 34:626–33. 10.1016/j.kjms.2018.05.008 30392569 PMC11915641

[56] Beg F Wang R Saeed Z Devaraj S Masoor K Nakshatri H. Inflammation-associated microRNA changes in circulating exosomes of heart failure patients. BMC Res Notes. (2017) 10:751. 10.1186/s13104-017-3090-y 29258606 PMC5735935

[57] Turchinovich A Weiz L Langheinz A Burwinkel B. Characterization of extracellular circulating microRNA. Nucleic Acids Res. (2011) 39:7223–33. 10.1093/nar/gkr254 21609964 PMC3167594

[58] Wu T Chen Y Du Y Tao J Li W Zhou Z. Circulating exosomal miR-92b-5p is a promising diagnostic biomarker of heart failure with reduced ejection fraction patients hospitalized for acute heart failure. J Thorac Dis. (2018) 10:6211–20. 10.21037/jtd.2018.10.52 30622793 PMC6297406

[59] Sen M Yasuhiko S Shinichiro S Daisaku N Masaya U Masahiko H. Circulating p53-responsive microRNAs are predictive indicators of heart failure after acute myocardial infarction. Circ Res. (2013) 113:322–6. 10.1161/CIRCRESAHA.113.301209 23743335

[60] Yang VK Loughran KA Meola DM Juhr CM Thane KE Davis AM. Circulating exosome microRNA associated with heart failure secondary to myxomatous mitral valve disease in a naturally occurring canine model. J Extracell Vesicles. (2017) 6:1350088. 10.1080/20013078.2017.1350088 28804599 PMC5533140

[61] Jansen F Yang X Proebsting S Hoelscher M Przybilla D Baumann K. MicroRNA expression in circulating microvesicles predicts cardiovascular events in patients with coronary artery disease. J Am Heart Assoc. (2014) 3:e1249. 10.1161/JAHA.114.001249 25349183 PMC4338711

[62] Yuan Y Du W Liu J Ma W Zhang L Du Z. Stem cell-derived exosome in cardiovascular diseases: macro roles of micro particles. Front Pharmacol. (2018) 9:547. 10.3389/fphar.2018.00547 29904347 PMC5991072

[63] Yang PC. Induced pluripotent stem cell (iPSC)-derived exosomes for precision medicine in heart failure. Circ Res. (2018) 122:661–3. 10.1161/CIRCRESAHA.118.312657 29496797 PMC5836744

[64] Wang X Gu H Qin D Yang L Huang W Essandoh K. Exosomal miR-223 contributes to mesenchymal stem cell-elicited cardioprotection in polymicrobial sepsis. Sci Rep. (2015) 5:13721. 10.1038/srep13721 26348153 PMC4562230

[65] Gray WD French KM Ghosh-Choudhary S Maxwell JT Brown ME Platt MO. Identification of therapeutic covariant microRNA clusters in hypoxia-treated cardiac progenitor cell exosomes using systems biology. Circ Res. (2015) 116:255–63. 10.1161/CIRCRESAHA.116.304360 25344555 PMC4338016

[66] Feng Y Huang W Wani M Yu X Ashraf M. Ischemic preconditioning potentiates the protective effect of stem cells through secretion of exosomes by targeting Mecp2 via miR-22. PLoS ONE. (2014) 9:e88685. 10.1371/journal.pone.0088685 24558412 PMC3928277

[67] Mayourian J Ceholski DK Gorski PA Mathiyalagan P Murphy JF Salazar SI. Exosomal microRNA-21-5p mediates mesenchymal stem cell paracrine effects on human cardiac tissue contractility. Circ Res. (2018) 122:933–44. 10.1161/CIRCRESAHA.118.312420 29449318 PMC5986183

[68] Khan M Nickoloff E Abramova T Johnson J Verma SK Krishnamurthy P. Embryonic stem cell-derived exosomes promote endogenous repair mechanisms and enhance cardiac function following myocardial infarction. Circ Res. (2015) 117:52–64. 10.1161/CIRCRESAHA.117.305990 25904597 PMC4482130

[69] Tang Y Huang Y Zheng L Qin S Xu X An T. Comparison of isolation methods of exosomes and exosomal RNA from cell culture medium and serum. Int J Mol Med. (2017) 40:834–44. 10.3892/ijmm.2017.3080 28737826 PMC5548045

